# Serum uric acid / serum creatinine ratio as a predictor of cardiovascular events. Detection of prognostic cardiovascular cut-off values

**DOI:** 10.1097/HJH.0000000000003319

**Published:** 2022-11-02

**Authors:** Edoardo Casiglia, Valérie Tikhonoff, Agostino Virdis, Guido Grassi, Fabio Angeli, Carlo M. Barbagallo, Michele Bombelli, Arrigo F.G. Cicero, Massimo Cirillo, Pietro Cirillo, Raffaella Dell’Oro, Lanfranco D’elia, Giovambattista Desideri, Claudio Ferri, Ferruccio Galletti, Loreto Gesualdo, Cristina Giannattasio, Guido Iaccarino, Luciano Lippa, Francesca Mallamaci, Stefano Masi, Alessandro Maloberti, Maria Masulli, Alberto Mazza, Alessandro Mengozzi, Maria Lorenza Muiesan, Pietro Nazzaro, Paolo Palatini, Gianfranco Parati, Roberto Pontremoli, Fosca Quarti-Trevano, Marcello Rattazzi, Gianpaolo Reboldi, Giulia Rivasi, Massimo Salvetti, Giuliano Tocci, Andrea Ungar, Paolo Verdecchia, Francesca Viazzi, Massimo Volpe, Claudio Borghi

**Affiliations:** a Studium Patavinum, Department of Medicine; b Department of Medicine, University of Padua, Padua; c Department of Clinical and Experimental Medicine, University of Pisa, Pisa; d Clinica Medica, Department of Medicine and Surgery, University of Milano-Bicocca, Monza; e Department of Medicine and Surgery, University of Insubria, Varese; f Biomedical Department of Internal Medicine and Specialistics, University of Palermo, Palermo; g Department of Medical and Surgical Science, Alma Mater Studiorum University of Bologna, Bologna; h Department of Public Health, “Federico II” University of Naples, Naples; i Nephrology, Dialysis and Transplantation Unit, Department of Emergency and Organ Transplantation, “Aldo Moro” University of Bari, Bari; j Department of Clinical Medicine and Surgery, “Federico II” University of Naples Medical School, Naples; k Department of Life, Health and Environmental Sciences, University of L’Aquila, L’Aquila; l Cardiology IV, “A.De Gasperi's” Department, Niguarda Ca’ Granda Hospital, Milan; m School of Medicine and Surgery, Milano-Bicocca University, Milan; n Department of Advanced Biomedical Sciences, “Federico II” University of Naples, Naples; o Italian Society of General Medicine (SIMG), Avezzano, L’Aquila; p CNR-IFC, Clinical Epidemiology of Renal Diseases and Hypertension, Reggio Cal Unit, Reggio Calabria; q Department of Internal Medicine, Santa Maria della Misericordia General Hospital, AULSS 5 Polesana, Rovigo; r Department of Clinical and Experimental Sciences, University of Brescia, Brescia; s Department of Medical Basic Sciences, Neurosciences and Sense Organs, University of Bari Medical School, Bari; t S. Luca Hospital, Istituto Auxologico Italiano & University of Milan-Bicocca, Milan; u Department of Internal Medicine, University of Genoa, and Policlinico San Martino, Genoa; v Medicina Interna I, Ca’ Foncello University Hospital, Treviso; w Department of Medical and Surgical Science, University of Perugia, Perugia; x Department of Geriatric and Intensive Care Medicine, Careggi Hospital and University of Florence, Florence; y Hypertension Unit, Division of Cardiology, Department of Clinical and Molecular Medicine, Faculty of Medicine and Psychology, University of Rome Sapienza, Sant’Andrea Hospital, Rome; z Hospital S. Maria della Misericordia, Perugia, Italy

**Keywords:** cardiovascular, creatinine, cut-off, epidemiology, uric acid

## Abstract

In the frame of the Uric Acid Right for Heart Health (URRAH) study, a nationwide multicenter study involving adult participants recruited on a regional community basis from all the territory of Italy under the patronage of the Working Group on Uric Acid and Cardiovascular Risk of the Italian Society of Hypertension, we searched for the cut-off values of the ratio between serum uric acid (SUA) and serum creatinine (sCr) able to predict cardiovascular (CV) events.

**Methods:**

Among 20 724 participants followed-up for 126 ± 64 months, after detecting cut-off by the receiver operating characteristic curves, we calculated by Cox models adjusted for confounders having CV events as dependent variable the hazard ratio (HR) of SUA/sCr > cut-off. We also verified if the role of cut-off varied with increasing SUA/sCr.

**Results:**

A plausible prognostic cut-off of SUA/sCr was found and was the same in the whole database, in men and in women (>5.35). The HR of SUA/sCr > cut-off was 1.159 (95% confidence interval [CI] 1.092–1.131, *P* < 0.03) in all, 1.161 (95% CI 1.021–1.335, *P* < 0.02) in men, and 1.444 (95% CI 1.012–1.113, *P* < 0.03) in women. In increasing quintiles of SUA/sCr the cut-offs were >3.08, >4.87, >5.35, >6.22 and >7.58, respectively. The HRs significantly increased from the 3rd to the 5th quintile (1.21, 95% CI 1.032–1.467, *P* = 0.018; 1.294, 95% CI 1.101–1.521, *P* = 0.002; and 1.642, 95% CI 1.405–1.919, *P* < 0.0001; respectively), that is, over 5.35, whereas the 2nd quintile was not significantly different from the 1st (reference).

**Conclusion:**

Having SUA/sCr >5.35 is an independent CV risk indicator both in men and women. The cut-off is dynamic and significantly increases with increasing SUA/sCr.

## INTRODUCTION

Uric acid is the final product of purine metabolism in humans. The association between serum uric acid (SUA) and cardiovascular (CV) disease has been investigated for almost 50 years [1] and the role of SUA as a CV risk factor and a metabolic mediator is growing. In the last years, the Uric Acid Right for Heart Health (URRAH) study [2] found univocal prognostic cut-off values of SUA able to predict incidence of CV events [3], myocardial infarction [4], stroke [5] and heart failure [6] also in type-2 diabetic patients [7] and in the aged [8].

SUA largely depends on renal function, but little is known about the prognostic value of SUA when it is indexed for renal function.

In addition to being generally accepted as another independent CV risk indicator [9], serum creatinine (sCr) is also an index of renal function, and can represent an easily available and inexpensive parameter for indexing SUA to renal function. As a matter of fact, the ratio between SUA_mg/dl_ and sCr_mg/dl_ is strictly associated to metabolic syndrome [10] and has sometimes been proposed as a metabolic mediator [11] and as a new variable able to explore CV risk [12] mainly in some categories of selected people such as patients with diabetes, nephropathy or chronic pulmonary disease [13–16] or in menopausal women [17].

However, curiously, no prognostic cut-off values have ever been determined for this a-dimensional variable.

The URRAH study was conceived and designed by the Working Group on Uric Acid and Cardiovascular Risk under the patronage of the Italian Society of Hypertension in order to ascertain the role of SUA as a risk factor in a nationwide, regional-based protocol involving a great number of Italian people [1,2]. One of the URRAH objectives is to find the cut-off values above which SUA and the variables derived from SUA, for instance the SUA/sCr ratio, entail an increased CV risk, stratifying participants into having or having not an increased probability of developing incident CV events during a long-lasting follow-up.

The present analysis intends to clarify: if the a-dimensional variable represented by the SUA_mg/dl_/sCr_mg/dl_ ratio is associated with CV risk, if a univariate cut-off level of SUA/sCr exists in men and women and can be confirmed as a risk indicator being accepted in multivariate Cox regression models adjusted for confounders, and if the cut-off values of SUA/sCr, if any, vary across sexes and with varying SUA/sCr value.

## METHODS

### Database and study protocol

The database called URRAH involves data on individuals aged 18–95 years collected on a regional community basis from all the territory of Italy with a median follow-up period of 133 months (interquartile range from 63 to 153 months) up to 31 July 2017. The study protocol has been previously extensively described [2–4]. In brief, a nationwide Italian database was built by collecting data on individuals from representative cohorts having SUA measurement and complete information about several variables including outcomes. A total of 20 724 participants were taken into account in the present analyses. For all participants, a standardized set of items was recorded, including demographics, anthropometric measures, metabolic parameters, smoking habit, systolic and diastolic arterial blood pressure, renal function, history of cardiovascular, renal and brain disease, concomitant treatments and outcomes. Hypertension was defined by the presence of at least two blood pressure recordings at least 140 or at least 90 mmHg or treatment with antihypertensive medications. Diabetes mellitus was defined if blood glucose was at least 126 mg/dl at fast or at least 200 mg/dl 2 h after 75 g oral glucose load or if glycated hemoglobin was >6%. Renal function was evaluated through sCr, and in sensitivity analysis through estimation of the glomerular filtration rate calculated according to the Chronic Kidney Disease Epidemiology Collaboration equation [18].

### Ethics

The study data were collected routinely or *ad hoc* in previously authorized studies. Participants underwent no extra tests or interventions, and there was no impact on participants’ care or outcome. The URRAH was performed according to the Declaration of Helsinki for Human Research (41st World Medical Assembly, 1990). The processing of the patients’ personal data collected in this study comply with the European Directive on the Privacy of Data. All data to be collected, stored and processed are anonymized, and all study-related documents are retained in a secure location. No personal information is stored on local personal computers. Approval was sought from the Ethical Committee of the Coordinating Center at the Division of Internal Medicine of the University of Bologna (No. 77/2018/Oss/AOUBo). Informed consent was obtained from all subjects at recruitment.

### Outcome

According to the URRAH protocol [3], incident events due to acute myocardial infarction, *angina pectoris*, heart failure, stroke, transient ischemic attack and hypertensive complications were taken into consideration during the follow-up (see Table 1s, Supplemental Digital Content for ICD10 codes). Events were double-checked with hospital and physicians’ files.

### Statistics

#### General description

The SAS package version 9.4 (SAS Institute, Cary, North Carolina, USA) was used for statistical analysis. A preliminary power analysis based on differences from stratified values of uric acid for *α* = 0.05 and power (1 − *β*) = 0.80 was performed. To our knowledge, no study exists about possible cut-off values of SUA/sCr discriminating participants into doomed to and not doomed to develop any CV event. Consequently, we considered 1 as a possible difference able to stratify participants according to the above-mentioned outcome. Power analysis showed that the number of participants in the database (*n* = 20 724) represented a sample largely sufficient to avoid *β* error. The Kolmogorov-Smirnov normality test was performed. Continuous variables were expressed as mean ± standard deviation and compared among classes or categories by the analysis of covariance adjusted time to time for proper confounders and followed by the Bonferroni's post hoc test. Categorical variables were compared by means of the Pearson *χ*^2^ test. In multivariate analyses, the covariables that were not independent from each other were previously log-transformed. The null hypothesis was rejected for values of *P* < 0.05.

The variable SUA/sCr was also divided into five increasing quintiles of 4145 participants each, to be used in descriptive comparative statistics by analysis of variance, and in multivariate Cox model as described below.

Estimated glomerular filtration rate (eGFR) was calculated [18] and used in sensitivity analysis as a putative further confounder in multivariate Cox models. The null hypothesis was rejected for values of *P* < 0.05.

#### Univariate prognostic cut-off values

The receiver operating characteristic (ROC) curves method was used to search for prognostic cut-off of SUA/sCr for CV events in the whole database and by sex. SUA/sCr was used as basic variable and CV events as dichotomic classification variable. The De Long *et al.* method [19] was used. Ratio of cases in the positive group (prevalence), sensitivity and specificity were calculated. ROC curves were generated in the whole database, and a prognostic cut-off value was identified as the curve point nearest to the 100% of axis of the ordinates [20]. In practical terms, this was made by identifying the SUA value associated to the highest values of the sum sensitivity + specificity. Youden's index [21] defined for all points of ROC curves was used as a criterion for selecting the optimum cut-off. The area under the curve was also shown for each ROC curves analysis [22].

#### Validation of the prognostic cut-off value and hazard ratio of being over cut-off

The cut-off values of SUA/sCr identified by mean of the ROC curves were used as independent variables in separate multivariate Cox analyses adjusted for the confounders already identified, having combined CV events as dichotomic dependent variable in the whole database. A cut-off value identified *via* the ROC curves method was considered as valid if accepted in the model being the null hypothesis rejected, otherwise it was considered a false cut-off. The corresponding hazard ratio with 95% confidence interval were obtained.

The validated cut-off values were used in the whole database, in the two sexes and in the five quintiles (see below) to stratify combined CV events in descriptive analysis and for generating outcome curves according to the Kaplan–Meier nonparametric estimator of limit product. Log-rank tests were used to assess differences between curves.

## RESULTS

### Descriptive statistics

The general characteristics of the 20 724 participants are shown in Table 1, also showing men and women separately. The characteristics of the five quintiles are shown in Table 2s, Supplemental Digital Content.

**TABLE 1:** General characteristics of the study participants also showing sex stratification

Variables	Whole database (*n* = 20 724)	Men (*n* = 10 229)	Women (*n* = 10 495)	*P* values between sexes
Age (years)	57.2 ± 14.7	56.5 ± 14.2	58.0 ± 15.4	<0.0001
Men (%)	49.4	–	–	–
SUA (mg/dl)	5.04 ± 1.39	5.26 ± 1.38	4.83 ± 1.38	<0.0001
sCr (mg/dl)	0.93 ± 0.25	0.96 ± 0.22	0.90 ± 0.28	<0.0001
SUA/sCr	5.56 ± 1.74	5.60 ± 1.76	5.51 ± 1.71	<0.0001
Smoking habit (yes %)	24.1	27.8	20.6	<0.0001
Ethanol intake (yes %)	62.6	64.8	60.4	<0.0001
Diabetes (yes %)	10.6	10.6	10.5	0.80 (NS)
Hypertension (yes %)	66.7	66.2	67.3	0.10 (NS)
Heart rate (bpm)	71.8 ± 12.3	70.7 ± 12.5	72.9 ± 11.9	<0.0001
Systolic BP (mmHg)	143.3 ± 2.8	143.0 ± 22.6	144.3 ± 24.9	<0.0001
Diastolic BP (mmHg)	85.4 ± 12.8	85.5 ± 12.5	85.3 ± 13.1	0.20 (NS)
BMI (kg/m^2^)	25.9 ± 4.2	26.6 ± 3.9	26.7 ± 4.6	0.03
LDLC (mg/dl)	135.6 ± 35.8	134.8 ± 35.9	135.1 ± 35.4	0.53 (NS)

BMI, body mass index; BP, arterial blood pressure; LDL-C, low-density-lipoprotein serum cholesterol; NS: no statistical difference; sCr, serum creatinine; SUA, serum uric acid.

During the 240-months follow-up, 2110 participants (10.2%) experienced a CV event, that is, 1030 men (10.1%) and 1080 women (10.3%, *P* = 0.6 between sex, NS).

### Search for cut-off value of serum uric acid/serum creatinine

**FIGURE 1:**
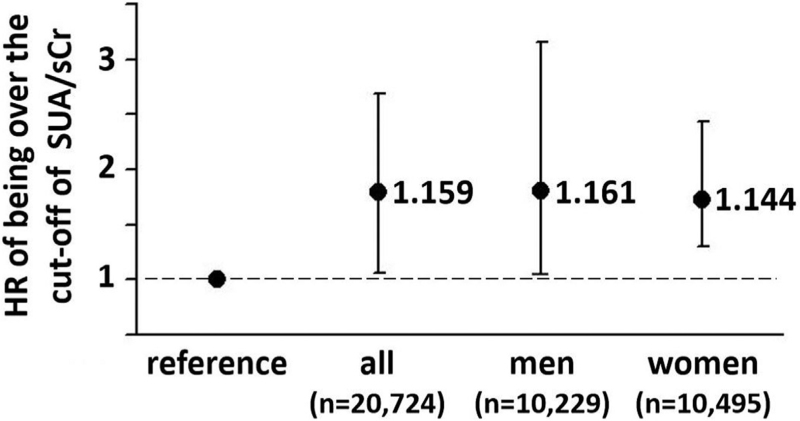
Hazard ratio (HR) of having SUA/sCr > cut-off in the whole database, in men and in women, after adjustment for age (years), sex when proper (1 = men, 0 = women), diabetes mellitus (1 = present, 0 = absent), smoking habit (1 = yes, 0 = no), arterial hypertension (1 = present 0 = absent) and alcohol consumption (1 = yes, 0 = no). Vertical bars represent the 95% CI. CI, confidence interval; sCr, serum creatinine; SUA, serum uric acid.

ROC curve furnished plausible univariate cut-off values of SUA/sCr for CV events (5.35), which was the same in the whole database, as well as separately in men and in women. The sex-specific ROC curves are shown in Figure 1s, Supplemental Digital Content, and the ROC parameters by sex in Table 3s, Supplemental Digital Content.

### Validation of cut-off values and hazard rations of being over serum uric acid/serum creatinine

**FIGURE 2:**
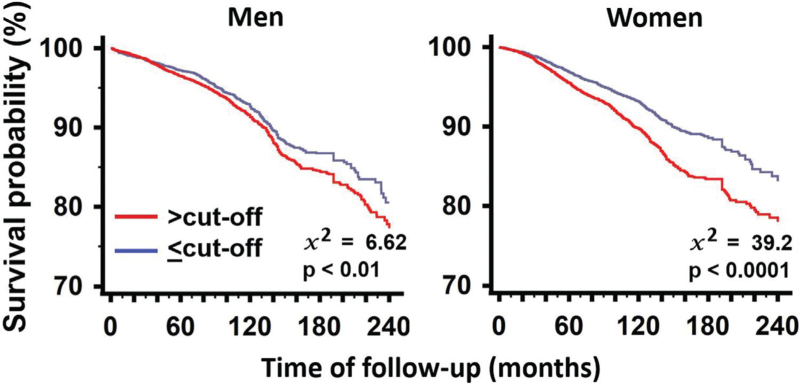
Kaplan–Maier curves of survival according to being or being not over the cut-off of SUA/sCr in men and women. NS: nonsignificant difference between sexes. Values of *P* indicate statistical difference vs. reference. NS: not significant. sCr, serum creatinine; SUA, serum uric acid.

After stratifying participants in those until the cut-off and those over the cut-off of SUA/sCr, multivariate Cox analyses adjusted for age (years), sex (1 = men, 0 = women), diabetes mellitus (1 = present, 0 = absent), smoking habit (1 = yes, 0 = no), arterial hypertension (1 = present 0 = absent) and alcohol consumption (1 = yes, 0 = no) demonstrate that being over the cut-off lead to higher risk both in men and in women (Fig. 1). The Cox analysis of the confounders is summarized in Table 4s, Supplemental Digital Content. In men or women, the curves of Kaplan-Mayer generated by being over the CU of SUA/sCr were superimposed (Figure 2s, Supplemental Digital Content).

Adding eGFR as further confounder did not change significantly the model although accepted as a direct predictive indicator in the whole database (hazard ratio [HR] 1.002, 95% confidence interval [CI] 0.001–0.003, *P* < 0.0001) and in women (HR 01.004, 95% CI 1.003–1.005, *P* < 0.0001).

Kaplan–Meier curves after stratification according to cut-off of SUA/sCr are shown in Fig. 2. The curves of participants having SUA ≤ cut-off and SUA > cut-off were clearly separate both in men and women.

### Analysis of the quintiles of serum uric acid/serum creatinine

**FIGURE 3:**
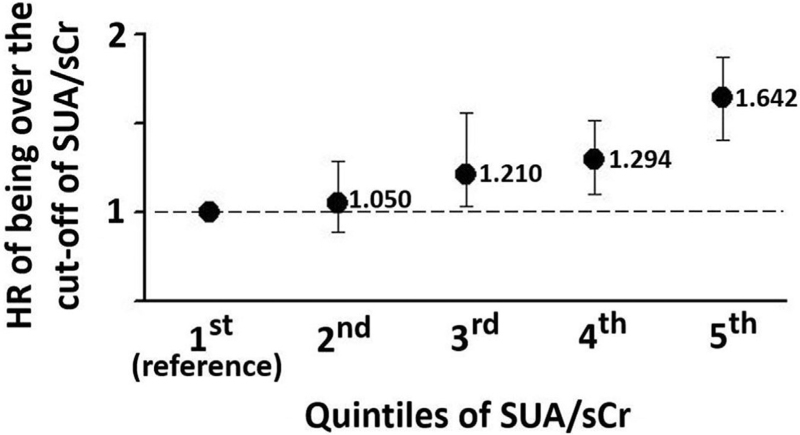
HRs of having SUA/sCr>cut-off in the quintiles of SUA/sCr. The 1^st^ quintile is the reference. The 3rd, 4th and 5th quintiles are different from reference. sCr, serum creatinine; SUA, serum uric acid.

In the five quintiles of SUA/sCr, the univariate putative prognostic cut-off values of SUA/sCr in relation to CV events were progressively increasing (>3.08 in 1st quintile, >4.87 in the 2nd, >5.35 in the 3rd, >6.22 in the 4th, and >7.58 in the 5th). The results of the ROC curves and analyses are shown in Figure 3s, Supplemental Digital Content and in Table 5s, Supplemental Digital Content, respectively.

When these cut-off values were used in multivariate Cox analyses adjusted for confounders, being over the cut-off positively and increasingly predicted CV events in the 3rd (HR 1.21, 95% CI 1.032–1.467, *P* = 0.018), 4th (HR 1.294, 95% CI 1.101–1.521, *P* = 0.002) and 5th quintile (HR 1.642, 95% CI 1.405–1.919, *P* < 0.0001) using 1st quintile as reference, while the 2nd quintile of SUA/sCr had the same risk than the reference (Fig. 3). The value of SUA/sCr>5.35 corresponding to the 3rd quintile was the threshold over which being over the cut-off had an increasing prognostic role.

When eGFR was added to the analysis as a putative confounder it was rejected from the model.

**FIGURE 4:**
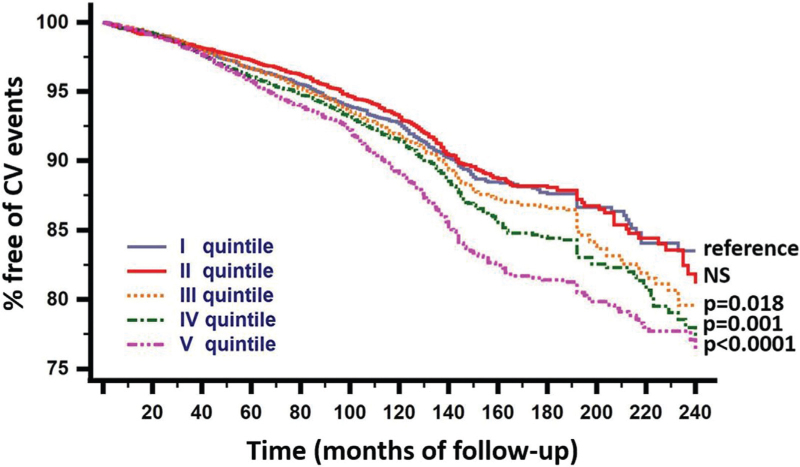
Kaplan–Maier curves of survival according to being or being not over the cut-off of SUA/sCr for each quintile of SUA/sCr. Values of *P* represent rejection of the null hypothesis vs. the reference. NS: nonsignificant difference. sCr, serum creatinine; SUA, serum uric acid.

Kaplan–Meier curves in after stratification according to cut-off of SUA/sCr in quintiles of SUA/sCr, in men and in women are shown in Fig. 4. The curves of participants having SUA/sCr ≤ cut-off and SUA/sCr > cut-off were clearly separate.

## DISCUSSION

First of all, the results of this present analysis confirm that SUA is an independent risk factor for CV events in a long follow-up also after indexing for renal function. This is consistent with a series of studies showing a positive association of incident CV events with SUA as a continuous variable, as well as with being over a well defined prognostic value of SUA [3–8].

This paper particularly takes into consideration the indexing of SUA to renal function represented by sCr. Since both SUA and sCr are expressed in the same unit of measurement (mg/dl), the SUA/sCr derivative variable is a-dimensional, that is purely numerical. SUA/sCr represents not only the result of an indexing but a novel item that can be treated in epidemiological setting as a new variable. We demonstrated SUA/sCr, when used as a continuous item, was a CV risk indicator both in men and women. This focused on the importance of taking into account renal function when using SUA as a risk factor in epidemiological setting. Neglecting this detail represents a severe bias and can lead to misleading results.

More importantly, up to date no prognostic cut-off values of SUA/sCr were known, that is, it was impossible to know if being over a certain value of SUA/sCr could lead to premature incident CV events. We demonstrated that precise cut-off values of SUA/sCr exist in the whole cohort, and separately in men and women. Participants being over the cut-off show higher prevalence of CV events during the follow-up. Not only this, but different (and increasing) cut-off exist with increasing SUA/sCr.

In the present analysis, the prognostic cut-off value of SUA/sCr able to identify the Participants at risk of developing premature CV events was more than 5.35 in the whole cohort, in men and in women: being over the cut-off, significantly led to HR >1 of developing a CV event in both sexes. This is undoubtedly the first report of a defined prognostic cut-off value of SUA predicting CV risk. Although it has been suggested that SUA is a better predictor of CV events than the SUA/sCr ratio [15], our protocol does not allow to clarify if there is any therapeutic implication to apply SUA/sCr rather than SUA for detection of CV risk in clinical setting.

On the contrary, this simply means that indexing for renal function is the right and necessary way to proceed when considering SUA a risk indicator, and that not indexing it to sCr, that is, not considering renal function, leads to falsely more optimistic results when using SUA as a CV prognostic marker.

Concerning sex, the literature suggests that a difference exists between men and women as regards SUA [1]. In our data, SUA/sCr >5.35 was the prognostic cut-off in the whole database, in men in women, confirming that, also after indexing for renal function, gender is a nonsignificant source of heterogeneity when SUA is considered as a risk factor.

In the continuation of the analysis, we wanted to see if SUA/sCr ratio was a constant value or if it varied with varying SUA/sCr. As a matter of fact, the cut-off values increased as the ratio increased, being higher in the higher than in the lower quintiles. Not only that, but when the cut-off values were used to divide, in every quintile, the participants destined and those not destined to develop a higher incidence of CV events in Cox models, we observed that the specific cut-off was valid only for the three higher quintiles, i.e. for values of SUA/sCr >5.35 (third quintile), while in the second quintile the risk was that of the first used as reference. In other words, being over the cut-off represents an increasing CV risk if the SUA/sCr ratio is over 5.35.

It is noteworthy that, when eGFR as a further marker of renal function was added to the Cox models having SUA/sCr as predictive variable, the results did not change significantly. This demonstrates that sCr is a sufficient and adequate indicator of the function of the kidneys. This is good, because sCr is easier to use in clinical practice and, unlike eGFR, it is directly measured variable, not calculated using other parameters according to empirical algorithms.

The main strength of the study described here is to have determined for the first time on a large population sample a prognostic cut-off of the SUA/sCr variable capable of predicting the incidence of cardiovascular events, and to have shown that it is dynamic and increases when SUA/sCr increases. A weakness of the study is that it did not take into account the eco-genetic context [23], which will have to be considered in subsequent studies, and that the URRAH is not strictly a population-based study, but rather a cohort study of data collected on a regional basis in the entire Italian territory. On the other hand, the collected database represents the largest number of Italian cases ever collected, and to our knowledge there is no one more representative of the Italian situation. Furthermore, the present analysis was limited to Italian people and its results cannot be directly applied to other ethnicities. In Chinese [24,25] and Japanese people [26], a simple association between the continuous variable SUA/sCr ratio and other CV risk factors was found, and Mazidi *et al.*[12], in a paper then withdrawn, described in the United States an association between the ratio as a continuous variable and mortality. On the other hand, to our knowledge, no paper defined cut-off values of the SUA/sCr ratio able to identify individuals at higher cardiovascular risk. A strength of our study is to have defined clear prognostic cut-off identified by the ROC curves methods and validated in multivariate models, able to stratify participants at higher CV risk.

In conclusion, the SUA/sCr ratio is more complete than SUA alone in predicting CV risk, as it includes the weight of renal function which is intimately linked to SUA. A clear and defined cut-off exists for SUA/sCr (>5.35), and in the frame of a nationwide study is able to stratify individuals destined to experience incident CV events during a long-lasting follow-up from those destined to be events-free. This cut-off, good for the whole cohort, is a dynamic value which tends to increase with increasing SUA/sCr when over 5.35. The SUA/sCr ratio is therefore qualifying itself as a low-cost reliable marker of CV risk and should be used systematically in epidemiological setting as well as in clinical practice to screen individuals at higher risk.

## ACKNOWLEDGEMENTS

Sources of Funding: none.

### Conflicts of interest

There are no conflicts of interest.

## Supplementary Material

Supplemental Digital Content
