# Supplementary material for: Serum uric acid / serum creatinine ratio as a predictor of cardiovascular events. Detection of prognostic cardiovascular cut-off values
Source: J Hypertens. 2022 Nov 2;41(1):180–6. doi: 10.1097/HJH.0000000000003319 (PMC9794153; doi:10.1097/HJH.0000000000003319)
Supplement: Supplemental Digital Content [file jhype-41-180-s001.doc]

**SERUM URIC ACID / SERUM CREATININE RATIO AS A PREDICTOR**

**OF CARDIOVASCULAR EVENTS. DETECTION OF PROGNOSTIC CUT-OFF VALUES**

**IN THE URRAH (URIC ACID RIGHT FOR HEART HEALTH) STUDY.**

Edoardo Casiglia^a^*, Valérie TIKHONOFF^b^*, Agostino VIRDIS^c^, Guido GRASSI^d^,

Stefano MASI^c^, Carlo M. BARBAGALLO^e^, Michele BOMBELLI^d^, Berardino BRUNO^f^,

Arrigo F. G. CICERO^g^, Massimo CIRILLO^h^, Pietro CIRILLO^i^,

Giovambattista DESIDERI^f^, Lanfranco D'ELIA^j^, Claudio FERRI^f^,

Ferruccio GALLETTI^j^, Loreto GESUALDO^i^, Cristina GIANNATTASIO^k,l^,

Guido IACCARINO^m^, Luciano Lippan, Francesca MALLAMACI^o^,

Alessandro MALOBERTI^k,l^, Alberto MAZZA^p^, Maria Lorenza MUIESAN^q^,

Pietro NAZZARO^r^, Paolo PALATINI^a^, Gianfranco PARATI^s,t^,

Roberto PONTREMOLI^u^, Fosca QUARTI-TREVANO^d^, Marcello RATTAZZI^b,v^,

Giulia RIVASI^w^, Massimo SALVETTI^q^, Giuliano TOCCI^x,y^, Andrea UNGAR^w^,

Paolo VERDECCHIA^z^, Francesca VIAZZI^u^, Massimo VOLPE^x,y^,

Claudio BORGHI^g^, on behalf of the Working Group on Uric Acid and Cardiovascular Risk of the Italian Society of Hypertension (SIIA).

*the two authors contributed to the manuscript equally

**Supplemental material**

**Supplemental Tables**

**Table 1s.** ICD10 codes used for diagnosis of cardiovascular events.

| Major cardiovascular diseases | I00-I78 |
| --- | --- |
| Diseases of heart | I00-I78, I11, I13, I20-I51 |
| Hypertensive heart disease | I11, I13 |
| Ischemia heart disease | I20-I25 |
| Other diseases od heart | I00-I09, I26-I51 |
| Hypertension and hypertensive renal disease | I10, I12 |
| Cerebrovascular diseases | I60-I69 |

**Table 2.** General characteristics of the study participants stratified by quintiles of SUA/sCr.

| **Parameters** | **1^st^ quintile** | **2^nd^ quintile** | **3^rd^ quintile** | **4^th^ quintile** | **5^th^ quintile** | **p for trend** |
| --- | --- | --- | --- | --- | --- | --- |
| **SUA/sCr** | 3.7±0.5 | 4.7±0.2 | 5.4±0.2 | 6.2±0.3 | 7.9±2.1 | <0.0001 |
| **SUA (mg/dL)** | 3.7±0.9 | 4.5±0.9 | 5.0±0.9 | 5.6±1.0 | 6.4±1.3 | <0.0001 |
| **sCr (mg/dL)** | 1.02±0.42 | 0.96±0.18 | 0.93±0.17 | 0.91±0.17 | 0.83±0.17 | <0.001 |
| **Age (years)** | 55.9±14.9 | 56.0±14.6 | 56.2±14.7 | 57.4±14.9 | 59.0±14.5 | <0.0001 |
| **Males (%)** | 45.2 | 48.1 | 50.7 | 52.6 | 50.2 | <0.0001 |
| **Smoking (%)** | 24.6 | 24.7 | 25.3 | 24.0 | 22.2 | 0.012 |
| **Ethanol (yes %)** | 57.7 | 60.8 | 61.8 | 64.7 | 68.6 | <0.0001 |
| **Diabetes (%)** | 10.5 | 8.8 | 9.5 | 10.3 | 18.7 | <0.0001 |
| **Arterial HT (%)** | 62.3 | 61.9 | 65.5 | 68.7 | 75.2 | <0.0001 |
| **BMI (kg/m^2^)** | 25.3±3.9 | 25.1±3.9 | 25.5±4.1 | 27.1±4.2 | 28.1±4.6 | <0.001 |
| **LDLC (mg/dL)** | 131.9±34.4 | 134.8±35.3 | 137.3±36.1 | 133.7±35.5 | 133.6±36.9 | 0.002 |

**Table 3s.** ROC parameters of SUA/sCr ratio in men and women. The ROC curves are shown in Figure 2s.

| **Quintiles** | **AUC**  **(95% CI)** | **Z**  **statistics** | **Sensitivity**  **%** | **Specificity**  **%** | **Youden**  **index** | **Cut-off (95% CI)** |
| --- | --- | --- | --- | --- | --- | --- |
| Men | 0.52 (0.51-0.53) | 1.938 | 56.2 | 48.0 | 0.042 | >5.35 (3.73-6.17) |
| Women | 0.55 (0.54-0.56) | 5.733 | 51.7 | 57.9 | 0.091 | >5.35 (5.27-6.62) |

**Table 4s.** Cox analysis the confounders in models having cardiovascular events as dichotomic dependent variable, and SUA/sCr>cut-off as independent covariables. HR: hazard ratio; CI: confidence intervals. The HRs of SUA/sCr >cut-off are shown in Figure 1 in main text.

| **Item** | **Estimate (SE)** | **z** | **p value** | **HR (95% CI)** |
| --- | --- | --- | --- | --- |
| **Whole database** | | | | |
| Age | 0.068 (0.002) | 13.69 | **<0.0001** | 1.070 (1.066-1.075) |
| Male sex | 0.100 (0.047) | 2.124 | **0.034** | 1.105 (1.007-1.212) |
| Diabetes | 0.755 (0.055) | 13.69 | **<0.0001** | 2.128 (1.909-2.371) |
| Smoking | 0.112 (0.058) | 1.920 | 0.055 | 1.507 (1.349-1.682) |
| Ethanol intake | 0.410 (0.056) | 7.385 | **<0.0001** | 1.507 (1.349-1.683) |
| Arterial hypertension | 0.206 (0.063) | 7.285 | **0.001** | 1.228 (1.085-1.391) |
| **Men** | | | | |
| Age | 0.062 (0.003) | 19.13 | **<0.0001** | 1.064 (1.058-1.070) |
| Diabetes | 0.772 (0.083) | 9.283 | **<0.0001** | 2.164 (1.973-2.374) |
| Smoking | 0.294 (0.077) | 3.843 | **<0.0001** | 1.341 (1.153-1.562) |
| Ethanol intake | 0.557 (0.083) | 6.686 | **<0.0001** | 1.745 (1.482-2.055) |
| Arterial hypertension | 0.160 (0.085) | 1.881 | 0.06 | 1.173 (0.993-1.387) |
| **Women** | | | | |
| Age | 0.074 (0.003) | 21.43 | **<0.0001** | 1.077 (1.070-1.083) |
| Diabetes | 0.717 (0.074) | 9.695 | **<0.0001** | 2.048 (1.770-2.370) |
| Smoking | -0.114 (0.093) | -1.222 | 0.2 | 0.892 (0.743-1.072) |
| Ethanol intake | 0.227 (0.078) | 2.909 | **0.004** | 1.245 (1.076-1.462) |
| Arterial hypertension | 0.320 (0.097) | 3.309 | **0.001** | 1.377 (1.138-1.667) |

**Table 5s.** ROC parameters in the five quintiles of SUA/sCr ratio.

| **Quintiles** | **AUC**  **(95% CI)** | **Z**  **statistics** | **Sensitivity**  **%** | **Specificity**  **%** | **Youden**  **index** | **Cut-off (95% CI)** |
| --- | --- | --- | --- | --- | --- | --- |
| 1^st^ | 0.52 (0.51-0.54) | 1.245 | 20.0 | 87.8 | 0.078 | >3.08 (2.69-4.00) |
| 2^nd^ | 0.51 (0.49-0.52) | 0.580 | 29.0 | 75.3 | 0.043 | >4.88 (4.57-4.99) |
| 3^rd^ | 0.52 (0.51-0.54) | 1.578 | 58.7 | 47.6 | 0.063 | >5.35 (5.08-5.49) |
| 4^th^ | 0.52 (0.51-0.54) | 1.540 | 47.2 | 59.4 | 0.067 | >6.22 (6.16-6.54) |
| 5^th^ | 0.52 (0.51-0.54) | 1.707 | 50.3 | 56.4 | 0.067 | >7.59 (7.20-9.34) |

**Supplemental Figures**

**
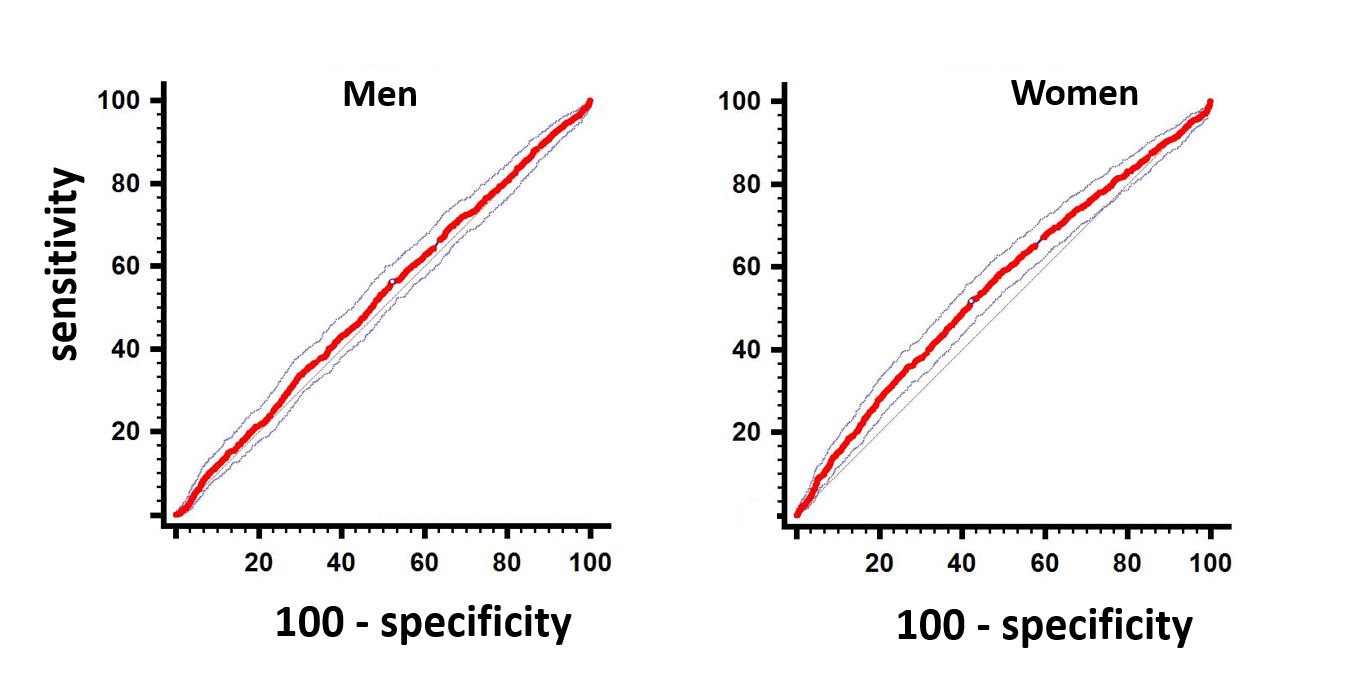
**

**Figure 1s.** ROC curves detecting the prognostic cut-off of SUA/sCr in men and women. ROC parameters are shown in Table 3s.

**
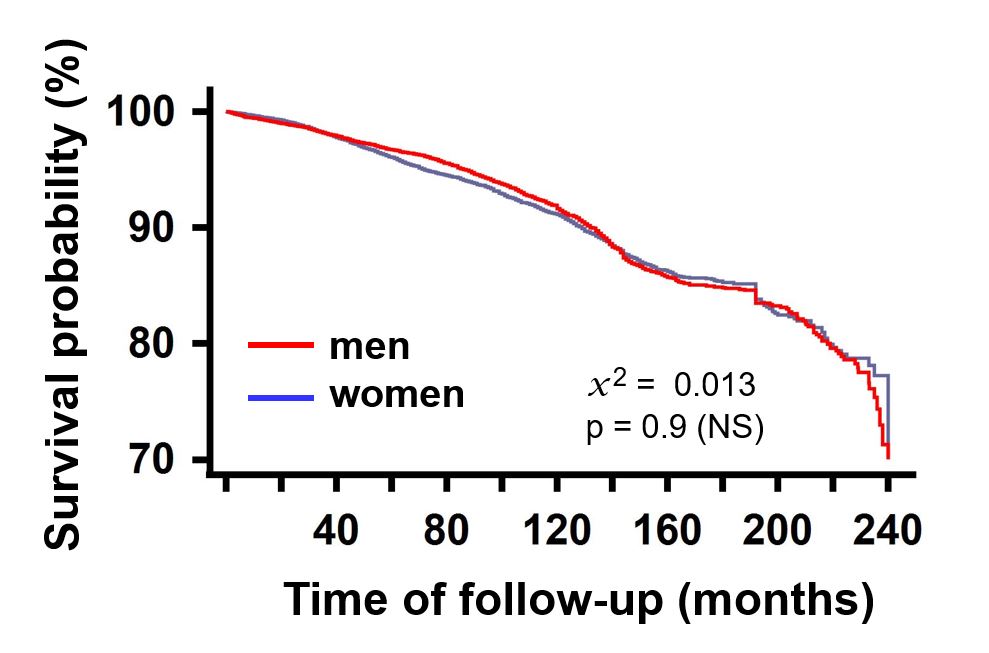
**

**Figure 2s.** Kaplan-Meier according to the cut-off value of SUA/sCr ratio. Being over the cut-off (>5.35) produced superimposed curves in the two sexes.


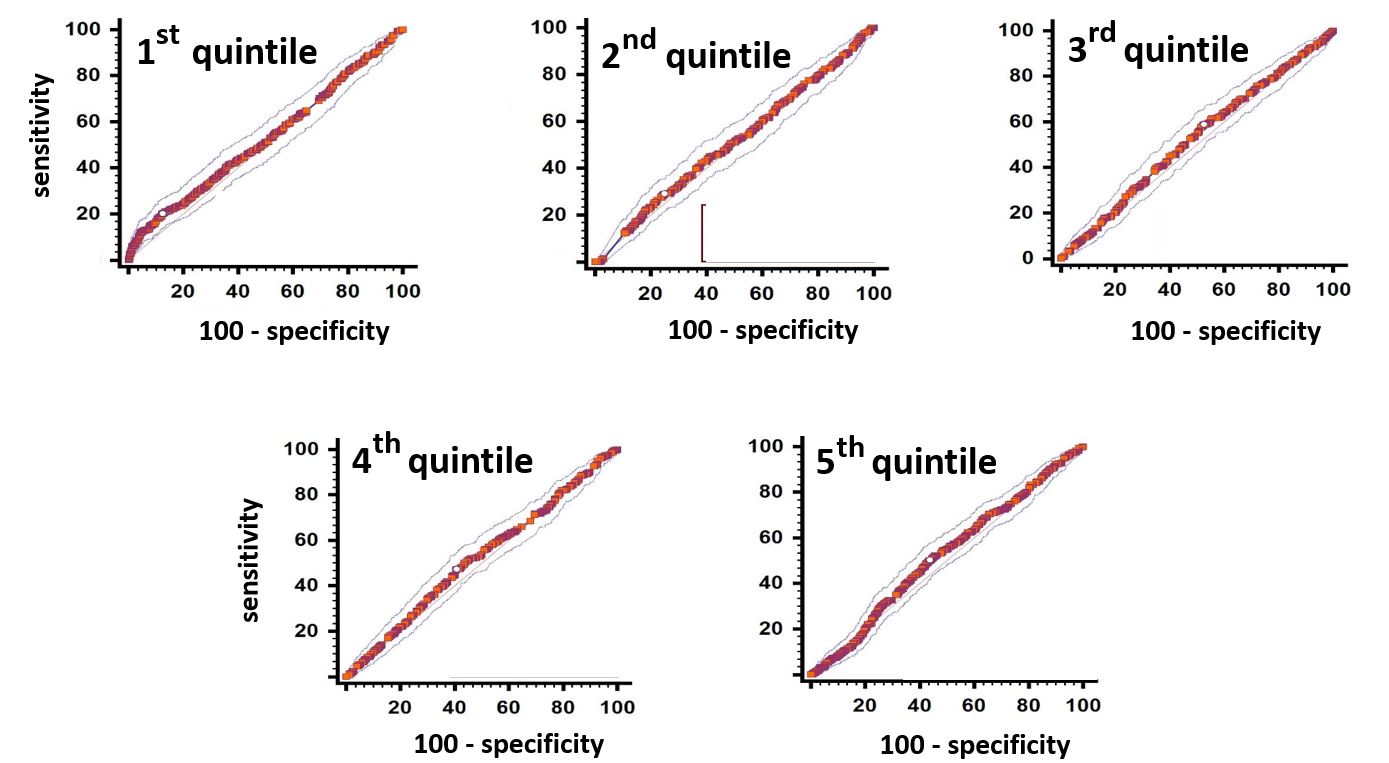


**Figure 3s.** ROC curves detecting the prognostic cut-off of SUA/sCr in the five quintiles of SUA/sCr. ROC parameters are shown in Table 5s.
